# The determination of lycopene *Z*‐isomer absorption coefficient on C30‐HPLC

**DOI:** 10.1002/fsn3.1879

**Published:** 2020-10-13

**Authors:** Jin Huang, Bodi Hui

**Affiliations:** ^1^ Department of Food Science Beijing Union University Beijing China; ^2^ COFCO Nutrition and Health Research Institute Co., Ltd. Beijing China

**Keywords:** all *E*‐isomer, evaporative light scattering detector, lycopene, specific absorption coefficient, *Z*‐isomer

## Abstract

Both *E*‐ and *Z*‐isomers of lycopene are encountered in nature. Although they were separated on C30‐HPLC by the mobile phase consisting of CH_3_CN‐MeOH (A) and MTBE (B), the quantification of *Z*‐isomers cannot be archived at present because either their commercially available reference samples are currently lacking, or they are unstable. In this study, both the specific and molar absorption coefficients of 5, 9, and 13 *Z*‐isomers in the mobile phase were determined on the analytical C30‐HPLC‐PDA‐ELSD, and further verified on the preparative C30‐HPLC‐PDA. The specific and molar absorption coefficients of 5, 9, and 13 *Z*‐isomers were finally verified to be $A_{1\text{cm}}^{1\%}$ = 3,422 and *ε*_mol_ = 183,717, $A_{1\text{cm}}^{1\%}$ = 2,183 and *ε*_mol_ = 117,199, and $A_{1\text{cm}}^{1\%}$ = 1,119 and *ε*_mol_ = 60,076, respectively, in the mobile phase. With these determined coefficients, the quantifications of 5, 9, and 13 *Z*‐isomers were able to be archived on C30‐HPLC‐PDA with the mobile phase applied in this investigation.

## Graphical abstract

This is the first study reporting the values of 5, 9, and 13 Z‐isomers of lycopene. It revealed that the absorption coefficient of lycopene isomer corresponded to the location of geometrical isomerization in the molecule. The specific and molar absorption coefficients of 5, 9, and 13Z‐isomers determined in this study have great potential to be applied in the Z‐isomer quantification in the fields of life sciences, food quality control, animal and human metabolism research, and pharmacology.

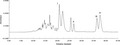

## 1 INTRODUCTION

Lycopene is a naturally occurring carotene composed solely of carbon and hydrogen atoms with the semisystem nomenclature of, *ψ*‐*ψ*carotene, molecular formula of C_40_H_56_, and relative molecular weight of 536.87 Da. Lycopene can be biologically synthesized as a natural product in higher plants and microorganisms, and chemically synthesized as a synthetic product. Both of them have been demonstrated to exhibit various health functions in vivo (Costa‐Rodrigues, Pinho, & Monteiro, 2020; Mein, Lian, & Wang, 2008; Wang, 2012). It is therefore considered to be an important nutrient or food functional factor. Its conjugated polyene chain, including 13 double bonds, allows lycopene to form multiple geometrical isomers including all *E*‐ and *Z*‐isomers. In a synthetic product, nearly all molecules exist in all *E*‐form. In natural product, most molecules exist in all *E*‐form, while the certain amount of the molecules exists in *Z*‐form, which is most commonly encountered as 5, 9, and 13 *Z*‐isomers.

Because *Z*‐isomers have biological functions and activities other than all *E*‐isomer in vivo, *Z*‐isomers have attracted a great deal of research interest in recent years (Fröhlich, Kaufmann, Bitsch, & Böhm, 2006; Graham, Carail, Caris‐Veyrat, & Lowe, 2012). It is therefore essential for most analytical practices in both academic and industrial bases, especially in food quality control and nutritional metabolism research, to determine the geometrical isomer composition of lycopene. Additionally, the geometrical isomer composition of lycopene is an important basis to evaluate the quality and source of lycopene products.

The all *E*‐ and *Z*‐isomers of lycopene were able to be well separated on C30‐HPLC under the condition of binary gradient elution (Rivera & Canela‐Garayoa, 2012). One of the most commonly used mobile phases consists of acetonitrile (CH_3_CN), methanol (MeOH), or the mixture of them, as mobile phase A, and methyl tert‐butyl ether (MTBE) as mobile phase B. A linear gradient elution is usually undertaken in 30–35 min.

After separation, the identification and structure elucidation of all *E*‐ and *Z*‐isomer fractions is usually achieved by electron absorption spectroscopy (UV‐Vis) (Amorim et al., 2018; Murillo, 2018), mass spectroscopy (MS), nuclear magnetic resonance spectroscopy (NMR) (Hengartner, Bernhard, Meyer, Englert, & Glinz, 1992; Honda et al., 2015, 2020; Takehara, Kuwa, Inoue, Kitamura, & Honda, 2015; Takehara et al., 2013), circular dichroic (CD) spectrum, and infrared (IR) spectrum. With the aid of commercially available external and internal reference samples, the quantification of all *E*‐isomer is able to be archived either on a UV‐Vis spectrophotometer or on a C30‐HPLC equipped with photodiode array detector (PDA) (Cucu, Huvaere, Van Den Bergh, Vinkx, & Van Loco, 2012; Irakli, Chatzopoulou, Kadoglidou, & Tsivelika, 2016; Ishida, Ma, & Chan, 2001). In addition, according to Lambert–Beer's law, the quantification of all *E*‐isomer can be achieved from its UV‐Vis absorbance and specific absorption coefficient at its maximum absorption wavelength (*λ*_max_). This method is often employed to validate the purity of a lycopene reference sample with the majority of all *E*‐isomer in analytical practice (Bunghez, Raduly, Doncea, Aksahin, & Ion, 2011). It was observed that the specific absorption coefficient of all *E*‐isomer was varied in different mediums, including organic solvents and supercritical carbon dioxide fluid, in correlation with medium polarity (Britton, Liaaen‐Jensen, Pfander, & Basel, 2020; Takaichi, 2000). For example, the specific absorption coefficient $A_{1\text{cm}}^{1\%}$ of all *E*‐isomer in petroleum ether (BP: 60–90°C) is 3,450. Unfortunately, the specific absorption coefficients of *Z*‐isomers such as 5, 9, and 13 *Z*‐isomers, the most commonly encountered *Z*‐isomers in nature, have not yet been reported in any medium. Due to the lack of the commercially available products of 9 and 13 *Z*‐isomers, the quantification of *Z*‐isomers is currently not able to be archived either by UV‐Vis spectrophotometer or by C30‐HPLC‐PDA. Because 5 *Z*‐isomer is unstable, its quantification cannot be easily undertaken with commercially available products as an external reference. At present, in most food quality control and nutrition metabolism research practice, the specific and molar absorption coefficients of each *Z*‐isomer is often replaced by that of all *E*‐isomer for *Z*‐isomer quantification (Chanforan, Loonis, Mora, Caris‐Veyrat, & Dufour, 2012; Mitrowska, Vincent, & von Holst, 2012; Pék, Daood, Lugasi, Fenyvesi, & Helyes, 2014). Under such circumstance, analytical error was significant.

This study aimed to determine the specific absorption coefficients ($A_{1\text{cm}}^{1\%}$ and *ε*_mol_) of lycopene 5, 9, and 13 *Z*‐isomers in HPLC mobile phase consisting of CH_3_CN, MeOH, and NTBE, a commonly employed mobile phase in lycopene geometrical isomer separation on C30 stationary phase. The determined coefficients can be applied to assess the amounts of those *Z*‐isomers on C30‐HPLC‐PDA with the mobile phase applied in this investigation.

## 2 MATERIALS AND METHODS

### 2.1 Materials and apparatus

The all *E*‐isomer sample of lycopene was kindly provided by Xinjiang TomatoRed Biotec. Co., Ltd., China. The sample was extracted and purified from tomato fruit. Its total lycopene amount is more than 98% (W/W) in which the total *E*‐isomer amount exceeds 97% (W/W). The sample was purified by an active alumina column to remove peroxides prior to use. The purity of the sample was then checked by C18‐HPLC before applied for UV‐Vis assay. The isomer composition of the sample was examined by C30‐HPLC before HPLC analysis.

Iodine, dichloromethane (DCM), n‐hexane, and petroleum (Pet.) ether (BP: 60–90°C) were of AR grade and from Beijing Chemical Co. CH_3_CN, MeOH, and MTBE used for HPLC mobile phase were chromatographic reagents and from Dikma Technologies, USA. C18 chromatographic stationary phase (open column packing materials) for the removal of iodine from geometrical isomer mixture was purchased from MERCK (C/N: 58,419) with 12 µm particle diameter.

HPLC used in this investigation was a gradient system from Waters, mainly consisting of a Waters Model 600 E solvent delivery system, a Waters Model 2,996 detector (PDA) with a quartz analytical flow cell and a quartz preparative flow cell, and a Waters Model 2,420 detector (ELSD) with a data processing unit. A PDA and ELSD were configured online in series to provide both electronic absorption spectral characteristics and mass of each fraction. The analytical HPLC column used for lycopene isomer separation was C30 YMC^TM^ Carotenoid S‐5 (4.6 mm × 250 mm, 5 μm). The preparative HPLC column used for the isomer fraction collection was a C30 YMC^TM^ Carotenoid (20 mm × 250 mm, 5 μm). The reproducibility of both analytical and preparative HPLC systems was carefully examined prior to be employed.

The UV‐Vis spectrophotometer with a 1 × 1×4 cm quartz curette was MultiSpec‐1501 and from SHIMADZU. It was carefully calibrated before being employed to minimize analytical error. The mass spectrometer was TRACE MS system and from Thermo Finnigan, USA. The rotary evaporator was Rotavapor® R‐100 and from BUCHI. The balance was XP205 (0.00001 g) and from METTLER TOLEDO.

### 2.2 Methods

#### 2.2.1 All *E*‐isomer absorption coefficient determination

5.00 mg lycopene all *E*‐isomer was (accurately to 0.00001 g) weighed and then well dissolved in 2 ml DCM in a 50‐ml brown volumetric flask. A stock solution was prepared by diluting the all *E*‐isomer and DCM solution to 50 ml with the HPLC mobile phase consisting of CH_3_CN, MeOH, and MTBE (33.75:11.25:55, V/V/V). A working solution was consequently prepared by diluting 1 ml of the stock solution to 100 ml with the mobile phase in a brown volumetric flask. The electronic absorption spectrum of the working solution was acquired in the range of 320–580 nm on the UV‐Vis spectrophotometer with the mobile phase as blank. The absorbance was measured at its *λ*_max_. The specific absorption coefficient $A_{1{\text{cm}}_{\text{AllE}}}^{1\%}$ of all *E*‐isomer was calculated as follows:

(1) $$A_{1{\text{cm}}_{\text{AllE}}}^{1\%}=\frac{\text{Ay}}{100x}$$

where

$A_{1{\text{cm}}_{\text{AllE}}}^{1\%}$ = all *E*‐isomer specific absorption coefficient, defined as the theoretical absorbance of 1% all *E*‐isomer solution in a 1 cm path‐length cuvette.

x = all *E*‐isomer amount[g].

y = volume of sample solution[ml].

A = absorbance of sample solution.

The molar absorption coefficient *ε*_mol_ of all *E*‐isomer was then calculated according to equation as below:

(2) $$\varepsilon_{\text{mol}}=\frac{A_{1\text{cm}}^{1\%}\times 536.87}{10}$$

where

*ε*_mol_ = molar absorption coefficient, defined as the theoretical absorbance of 1 M concentration solution in a 1 cm path‐length cuvette.

$A_{1\text{cm}}^{1\%}$= specific absorption coefficient, defined as the theoretical absorbance of 1% concentration solution in a 1 cm path‐length cuvette and calculated according to Equation (1).

536.87 = lycopene molecular weight.

#### 2.2.2 Geometrical isomer mixture preparation

700 mg of all *E*‐isomer was accurately (to 0.00001 g) weighed and dissolved in 200 ml DCM. 500 mg I_2_ was accurately weighed (to 0.00001 g) and dissolved in 100 ml DCM. The two solutions were well mixed and immediately irradiated at 40 cm from a 60 W fluorescent lamp for 30 min at room temperature to induce the geometrical isomerization of all *E*‐isomer. The reaction was stopped by the removal of DCM under the conditions of −0.085 MPa, 35°C, and 30 rpm on rotary evaporator. The reaction product was redissolved in 20 ml DCM. A bulk of C18 stationary phase was prepared to pack an open chromatography column (1 cm id. and 10 cm bed height). The 20 ml reaction product solution was consequently loaded on the column and eluted with 1,000 ml Pet. ether at room temperature. Under such chromatographic conditions, mixed lycopene geometric isomers were eluted out to form a single fraction. The fraction was collected and dried at −0.085 MPa, 35°C, and 30 rpm on rotary evaporator. Any light was strictly avoided in whole operation. After the chromatographic purification, most of I_2_ was removed from the reaction product. The I_2_‐removed isomer mixture was stored under nitrogen at −80°C in dark. 0.1 and 0.5 mg/ml I_2_‐removed isomer mixture were prepared as stock solution by DCM for analytical and preparative HPLC separations, respectively. Both of the solutions were filtered by 0.45μ filter before injection.

#### 2.2.3 Isomer separation and fraction identification on analytical C30‐HPLC

Lycopene geometrical isomers were well separated from the mixture on C30 YMC Carotenoid S‐5 (4.6 × 250 mm, 5 μm) column under the conditions of CH_3_CN‐MeOH (75:25, V/V) as mobile phase A and MTBE as mobile phase B. A linear binary gradient elution was undertaken with an increased B from 0% to 55% (V/V) in 8 min, followed by an isocratic elution of 55% B for another 27 min (for 35 min in total), at the flow rate of 1.0 ml/min and with the injection volume of 20 μl. The column temperature was maintained at 25°C.

With the aid of online equipped PDA, all *E*‐isomer fraction from the mixture was preliminary identified by comparing its chromatographic behavior, such as retention time, and electronic absorption spectral characteristics such as *λ*_max_, with those of reference samples to the all *E*‐isomer sample before iodine is induced. The *Z*‐isomer fractions, including 5, 9, and 13 *Z*‐isomers, were identified in comparison with their reported chromatographic behaviors, such as retention time, and electronic absorption characteristics, mainly including *λ*_max_, the absorption intensity ratio of *Z*‐isomers, and main absorption bands.

The mass spectrum of each fraction was also acquired under the conditions of direct sample insertion, electron impact (EI) ionization at 240°C source temperature, 200 mA emission current and 70 eV electron energy, 50–750 Da(*m/z*) scanning range, and 4‐s scanning time.

#### 2.2.4 Dependence of peak area upon fraction amount from PDA and ELSD

The 0.1 mg/ml stock solution was half–half diluted for 7 times with DCM to produce 8 working solutions at the concentrations of 100, 50, 25, 12.5, 6.25, 3.125, 1.5625, and 0.78125 μg/ml (in terms of all *E*‐isomer). Each solution was then injected to C30‐HPLC‐PDA‐ELSD. All E‐, and 5, 9, and 13 *Z*‐isomers were well separated under the conditions given in Section 1.2.3.

The chromatographic profile of each sample was acquired at 472 nm, all *E*‐isomer λ_max_, while the electronic absorption spectrum of each fraction was acquired in the range of 320–580 nm on PDA. After the electronic absorption spectral characteristics of each *Z*‐isomer fraction were measured, the peak area of each *Z*‐isomer fraction was integrated on chromatogram acquired at the *λ*_max_ of each fraction. The chromatogram of each sample on ELSD was acquired under the conditions of 30 psi carrier gas pressure, 40°C nebulizer temperature, 45°C drift tube temperature, and the gain of 1. The peak area of each fraction was then integrated. A correlation of sample amount with peak area was linearly regressed of each isomer from PDA and ELSD, respectively. The R^2^ value of each regression was given to verify the linearity of each sample amount and peak area correlation.

#### 2.2.5 Dependence of peak area from PDA upon that from ELSD

A fraction peak area correlation of all *E*‐isomer, and 5, 9 and 13 *Z*‐isomers acquired between PDA and ELSD was further linearly regressed. The R^2^ value of each regression was calculated and expressed to verify the linearity of each correlation. The slope (*K*_PDA‐ELSD_) of each regression was calculated and expressed.

#### 2.2.6 *Z*‐isomer absorption coefficient calculation on analytical C30‐HPLC

The specific absorption coefficient of each *Z*‐isomer is theoretically calculated according to that of all *E*‐isomer, the ratio of *Z*‐isomer slope (PDA peak area/ELSD peak area), and all *E*‐isomer slope (PDA peak area/ELSD peak area) as shown in Equation (3):

(3) $$A_{1{\text{cm}}_{\text{nZ}}}^{1\%}=\frac{A_{1{\text{cm}}_{\text{AllE}}}^{1\%}\times K_{{\text{nZ}}_{\text{PDA}\;‐\;\text{ELSD}}}}{K_{{\text{AllE}}_{\text{PDA}\;‐\;\text{ELSD}}}}$$

where

$A_{1{\text{cm}}_{\text{nZ}}}^{1\%}$ = *Z*‐isomer specific absorption coefficient. n is *Z*‐isomerization position, such as 5, 9, and 13.

$A_{1{\text{cm}}_{\text{AiiE}}}^{1\%}$ = All *E*‐isomer specific absorption coefficient and calculated according to Equation (1).

$K_{{\text{nZ}}_{\text{PDA}\;‐\;\text{ELSD}}}$ = *Z*‐isomer slope (PDA peak area/ELSD peak area). n is *Z*‐isomerization position, such as 5, 9, and 13.

$K_{{\text{AllE}}_{\text{PDA}\;‐\;\text{ELSD}}}$ = All *E*‐isomer slope (PDA peak area/ELSD peak area).

The molar absorption coefficient *ε*_mol_ of each *Z*‐isomer was then calculated according to Equation (2).

#### 2.2.7 Isomer fraction separation and identification on preparative C30‐HPLC

Each isomer fraction from the iodine‐induced lycopene geometrical isomer mixture was also readily separated on preparative C30‐HPLC‐PDA under the conditions of C30 YMC Carotenoid (20 mm × 250 mm, 5 μm) preparative column, CH_3_CN‐MeOH (75:25, V/V) as mobile phase A, and MTBE as mobile phase B. With a linear gradient elution, mobile phase B increased from 0% to 55% (V/V) in 8 min and kept at 55% (V/V) from 8 to 45 min. The flow rate was 7.0 ml/min. The sample injection volume was 1.0 ml. The PDA signal acquisition conditions were the same as those on the analytical HPLC. Each fraction was identified as same as that undertaken on analytical column. A 1.0 ml sample injection loop was used to replace that applied on analytical HPLC.

#### 2.2.8 Fraction peak area integration on preparative C30‐HPLC and amount assay

The iodine‐induced lycopene geometrical isomer mixture was repeatedly injected for 70 times to collect the fraction of each isomer until the quantity of each isomer was enough for gravimetric measurement. The fraction peak area of each isomer was integrated on the chromatogram acquired at the *λ*_max_ of the isomer at each injection. The peak area sum of each isomer was consequently counted for 70 injections. The fraction of each isomer was collected at each injection; that is, the fraction of each isomer was repeatedly collected 70 times. The mobile phase was removed from collected fractions on a rotary evaporator at −0.085 MPa, 4℃ (in ice bath), and 30 rpm after each collection. Light was strictly avoided in the whole operation. The total amount for 70 injections of each isomer was accurately (to 0.00001 g) weighed by balance.

#### 2.2.9 *Z*‐isomer absorption coefficient calculation on preparative C30‐HPLC

The specific value (SV_Peak area‐Amount_) of the peak area sum and the total amount of each isomer were calculated after 70 injections. The specific absorption coefficient of each *Z*‐isomer was calculated according to the equation as follows:

(4) $$A_{1{\text{cm}}_{\text{nZ}}}^{\prime 1\%}=\frac{A_{1{\text{cm}}_{\text{AllE}}}^{1\%}\times {\text{SV}}_{{\text{nZ}}_{\text{Peakarea}\;‐\;\text{Amount}}}}{{\text{SV}}_{{\text{AllE}}_{\text{Peakarea}\;‐\;\text{Amount}}}}$$

where

$A_{1{\text{cm}}_{\text{nZ}}}^{\prime 1\%}$ = *Z*‐isomer specific absorption coefficient. n is *Z*‐isomerization position, such as 5, 9, and 13.

$A_{1{\text{cm}}_{\text{AiiE}}}^{1\%}$ = all *E*‐isomer specific absorption coefficient and calculated according to Equation (1).

${\text{SV}}_{{\text{nZ}}_{\text{Peakarea}\;‐\;\text{Amount}}}$ = the specific value of the peak area sum and the total quantity of *Z*‐isomer fraction for 70 injections. n is *Z*‐isomerization position, such as 5, 9, and 13.

${\text{SV}}_{{\text{AllE}}_{\text{Peakarea}\;‐\;\text{Amount}}}$ = the specific value of the peak area sum and the total quantity of all *E*‐isomer fraction for 70 injections.

The molar absorption coefficient *ε*_mol_ of each *Z*‐isomer was then calculated according to Equation (2).

#### 2.2.10 Repeatability

All measurements undertaken in this study were repeated for more than 6 times. The RSD of each measurement was less than 0.7%.

## 3 RESULTS AND DISCUSSION

### 3.1 Determination of all *E*‐isomer absorption coefficients in C30‐HPLC mobile phase

In order to determine the specific absorption coefficients of *Z*‐isomers, the specific absorption coefficient of all *E*‐isomer was preliminarily measured in the mobile phase consisting of CH_3_CN, MeOH, and MTBE (33.75:11.25:55, V/V/V), which was the C30‐HPLC mobile phase composition when all *E*‐isomer passed through the flow cell of PDA on analytical C30‐HPLC. To ensure better reproducibility, the determination was undertaken on a UV‐Vis spectrophotometer in this investigation. Both sample and blank solvents used for the determination were the C30‐HPLC mobile phase. Data from this investigation suggested that the λ_max_ of all *E*‐isomer was at 472 nm, while the $A_{1\text{cm}}^{1\%}$ of all *E*‐isomer was 3,438; that is, the *ε*_mol_ of all *E*‐isomer was 187,260 in the mobile phase. In comparison with those in petroleum ether (λ_max_ = 474 nm and $A_{1\text{cm}}^{1\%}$ = 3,450), *E*‐isomer showed a slight (2 nm) hypsochromic shift at λ_max_ and a little (12) decrease of $A_{1\text{cm}}^{1\%}$ in the mobile phase. With regard to the previously reported absorption coefficient of all *E*‐isomer in different solvents, those observations were fundamentally due to variations in solvent polarity consider rephrasing to solvent species and concentration if a mixed solvent is applied.

### 3.2 Geometrical isomer separation on analytical C30‐HPLC‐PDA‐ELSD and fraction identification

**FIGURE 1:**
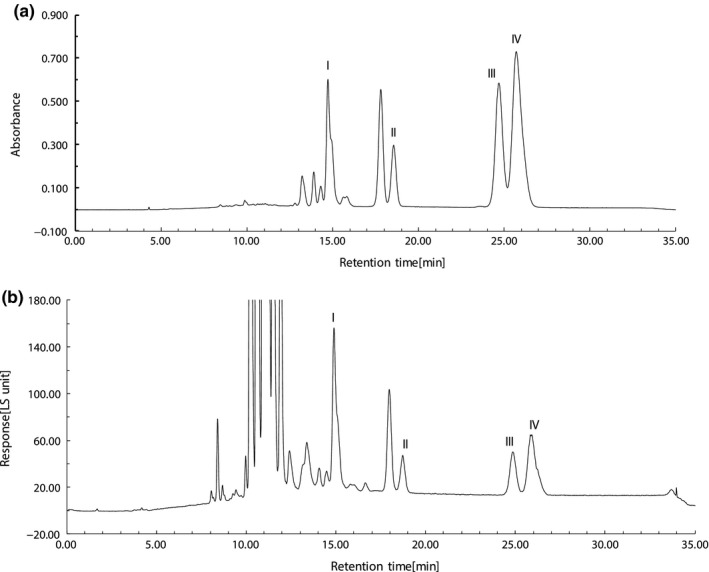
(a) The analytical C30‐HPLC profile of iodine‐induced lycopene geometrical isomer mixture from PDA. (b) The analytical C30‐HPLC profile of iodine‐induced lycopene geometrical isomer mixture from ELSD. HPLC conditions: as described in Section 1.2.3. Monitoring wavelength: 476 nm. Fraction identifications: I = 13 *Z*‐isomer; II = 9 *Z*‐isomer; III = all *E*‐isomer; IV = 5 *Z*‐isomer

The C30‐HPLC profiles from both PDA and ELSD of iodine‐induced lycopene geometrical isomer mixture are given in Figure 1a,b, respectively. Data from Figure 1a,b suggested that the lycopene geometrical isomer fractions were well separated under the HPLC conditions employed in this investigation. The all *E*‐isomer fraction was identified by comparing its retention time and electron absorption spectral characteristics with those of external reference sample. The 5, 9, and 13 *Z*‐isomer fractions were identified with reference to reported data on their chromatographic behaviors, for example, retention time, elution order, electronic absorption spectral characteristics such as *λ*_max_, absorption intensity ratio between *Z*‐peak and major absorption band, mass spectrum, etc.

**FIGURE 2:**
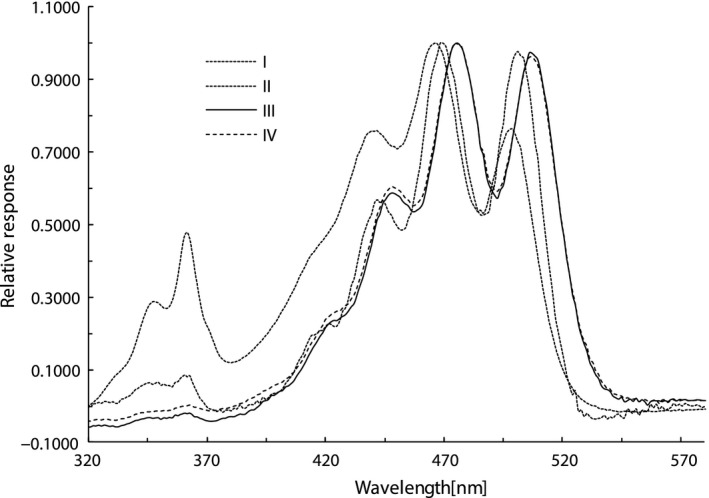
Electron absorption spectra of lycopene geometrical isomers (replotted from PDA acquisition data). Solvents: C30‐HPLC mobile phase at composition when corresponding isomer fraction passed through the flow cell of PDA

The electronic absorption spectra of each fraction are shown in Figure 2. In the HPLC mobile phase used in this investigation, it was observed that the major absorption band λ_max_ values of Fractions I, II, III, and IV were 468, 468, 475, and 475 nm, respectively, and the *Z*‐peak λ_max_ values of Fractions I, II, III, and IV were 362, 362, 363, and 363 nm, respectively. The λ_max_ hypsochromic shifts of three *Z*‐isomers were observed in comparison with those of all *E*‐isomer and indicated that the molecular 1^1^Ag—1^1^Bu transition energy was increased when *Z*‐isomerization took place in lycopene molecule.

A variation in the absorption intensity ratio of *Z*‐peak and major absorption band of each *Z*‐isomer is also seen from Figure 2 in comparison with that of all *E*‐isomer. The *Z*‐peak and major absorption band intensities at *max* are measured, respectively, from the absorbance at 550 nm as the baseline or zero value. The ratio of two measured values can be expressed as relative *Z*‐peak intensity [%]. It was seen that the relative *Z*‐peak intensities of Fractions I, II, III, and IV were 11.81, 11.19, 4.55, and 4.62[%].

Additionally, a variation in the fine electronic absorption spectral structure of each fraction can be observed in Figure 2. On the spectra, if the minimum between the right and middle peaks is set as the baseline or zero value, the right and middle peak heights can be designated as III and II, respectively. The III/II values of Fractions I, II, III, and IV are calculated to be 69.04, 68.04, 74.26, and 75.03[%].

**FIGURE 3:**
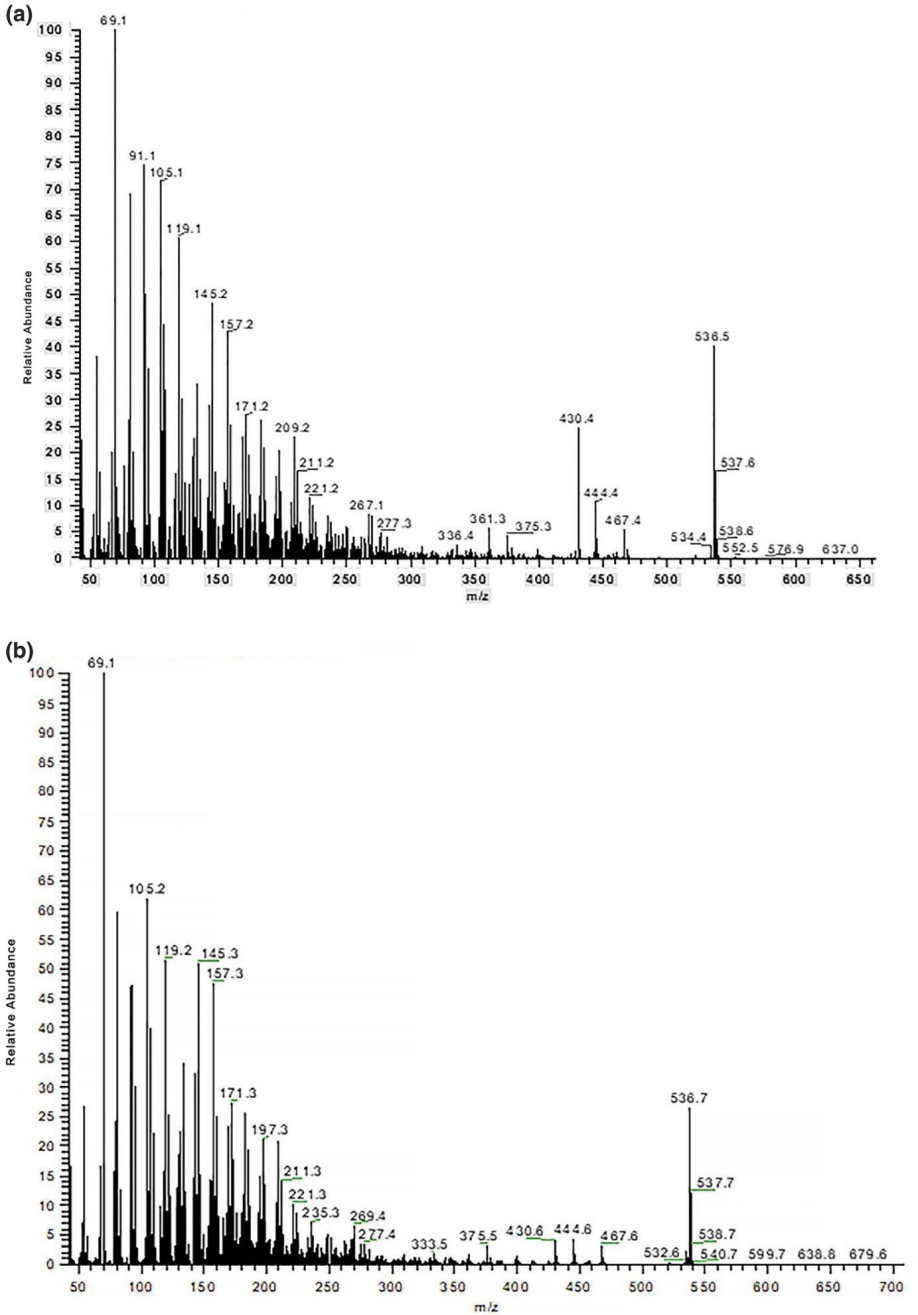
The mass spectra of fractions I (a) and III (b). MS conditions: as shown in Section 1.2.3. Data from the electronic absorption spectral and mass characteristics suggested that the identification of each fraction is: I = 13 *Z*‐isomer; II = 9 *Z*‐isomer; III = all *E*‐isomer; and IV = 5 *Z*‐isomer

It was observed that all fractions exhibited identical mass spectral characteristics, such as the parent ion at 536.5(*m/z*) and fragment at 69.1(*m/z*); *that is,* they had an identical molecule formula. The mass spectra of factions I and III are given in Figure 3. Fractions I, II, III, and IV are likely to be considered as the isomers of each other.

### 3.3 Dependence of peak area upon fraction amount from PDA and ELSD

It was observed in this investigation that the peak area was linearly and positively dependent on the amount of each isomer on PDA when the total lycopene concentration of the iodine‐induced isomer mixture varied from 100.00 (the stock solution concentration) to 0.78 μg/ml (the final working solution concentration after 128‐time dilution). The linearity of those regressions was examined and expressed as a *R* ^2^ value. Data from this investigation suggested that the *R* ^2^ values of *E*‐isomer, and 5, 9, and 13 *Z*‐isomers were all greater than or equal to 0.996. It was therefore confirmed that the peak area at λ_max_ over the electronic absorption band acquired in this study was linearly and positively dependent on the amount of each isomer on PDA equipped to HPLC, that is, followed Lambert–Beer's law.

**FIGURE 4:**
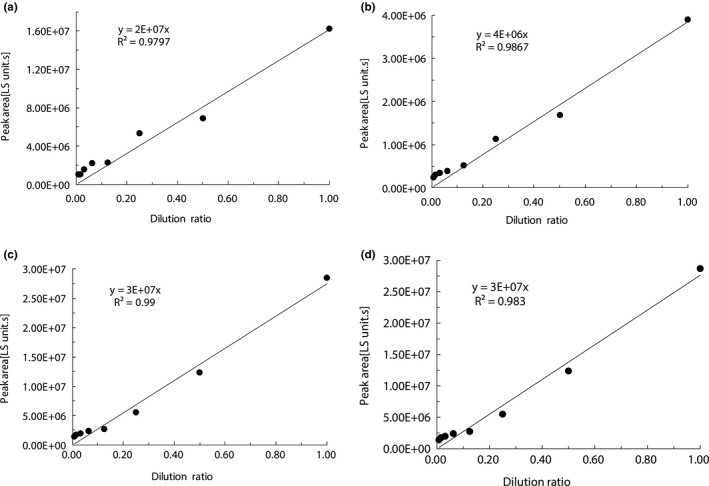
Dependence of peak area on fraction amount on ELSD. (I) Fraction I identified as 13 *Z*‐isomer; (II) Fraction II identified as 9 *Z*‐isomer; (III) Fraction III identified as all *E*‐isomer; (IV) Fraction identified as 5 *Z*‐isomer. HPLC conditions: as described in Section 1.2.3

Theoretically, the correlation of peak area with fraction amount is positive but may not be linear, and is likely to be a single logarithm or double logarithm on ELSD (Dvořáčková, Šnóblová, & Hrdlička, 2014; Elder, Borman, & Okafo, 2015; Zhang, Kurita, Venkatramani, & Russell, 2019). When sample amount is varied in a limited range, the correlation of peak area with fraction amount can be linearly regressed with an acceptable *R* ^2^. In this investigation, a linear dependence of peak area upon fraction amount of each isomer was regressed and expressed in Figure 4 (I), (II), (III), and (IV) when the total lycopene amount of the iodine‐induced isomer mixture was varied from 100.00 to 0.78 μg/ml on ELSD. The R^2^ values of all *E*‐isomer, and 5, 9, and 13 *Z*‐isomer were 0.9834, 0.983, 0.9757, and 0.9614, respectively, and greater than or equal to 0.9614.

### 3.4 Dependence of peak area from PDA upon that from ELSD

**FIGURE 5:**
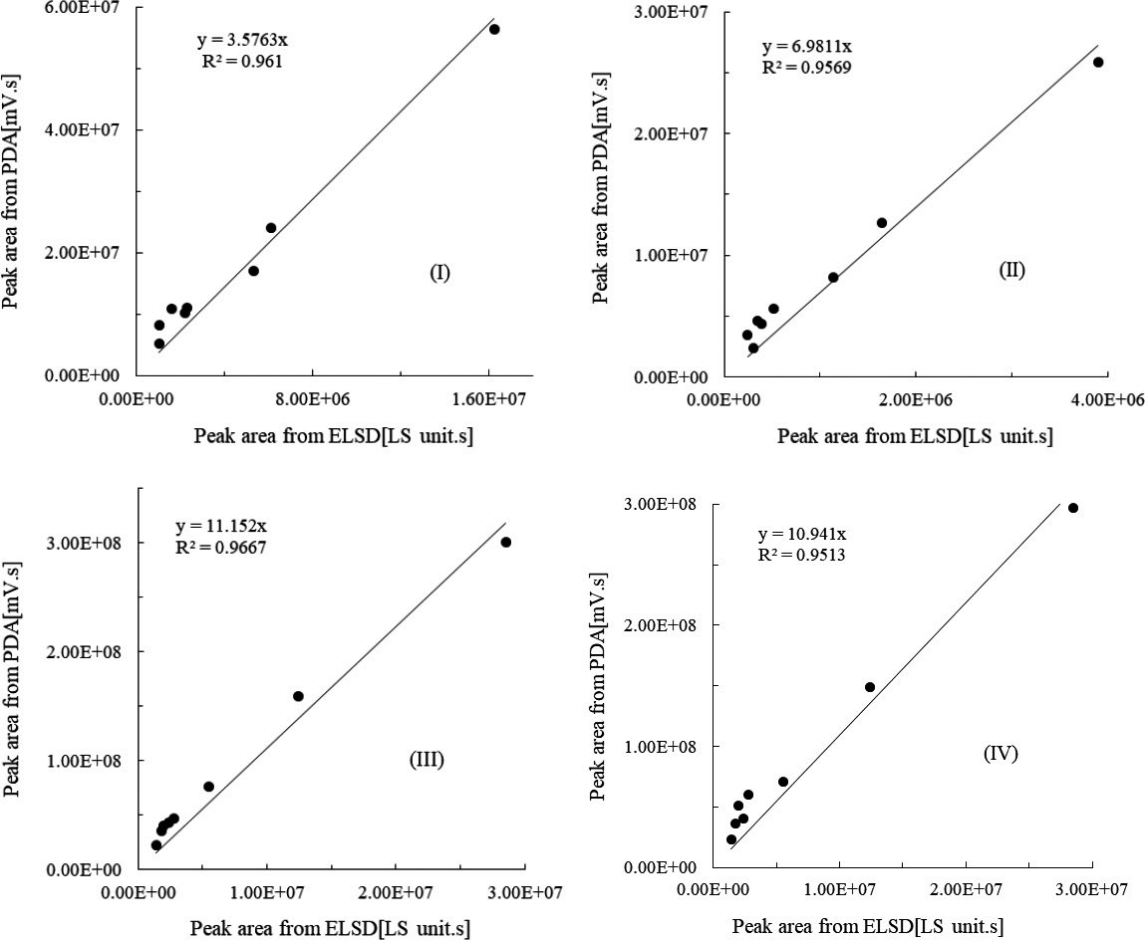
Dependence of peak area from PDA upon that from ELSD. (I) Fraction I identified as 13 *Z*‐isomer; (II) Fraction II identified as 9 *Z*‐isomer; (III) Fraction III identified as all *E*‐isomer; (IV) Fraction identified as 5 *Z*‐isomer. HPLC conditions: as described in Section 1.2.3

If the linear dependence of peak area on fraction amount on ELSD was workable, as shown in Figure 4, the linear dependence of peak area from PDA upon that from ELSD was able to be readily regressed of each isomer. A linear dependence of peak area from PDA upon that from ELSD of each isomer is given in Figure 5. The slope (*K_Z_*_n PDA‐ELSD_) and R^2^ value of the regression equation are given in Figure 5.

### 3.5 *Z*‐isomer absorption coefficient calculation on analytical C30‐HPLC

In a diluted solution, the specific absorption coefficient of all *E*‐isomer is defined as the specific value of all *E*‐isomer absorbance, usually shown as absorption intensity on spectrum, at its λ_max_ in ultraviolet and visible (UV‐Vis) region and fraction amount. The specific value is needed to be expressed together with a proper constant to form a coefficient such as specific absorption coefficient expressed as $A_{1\text{cm}}^{1\%}$ when 1% solution and 1 cm optical path were applied, and molar absorption coefficient (*ε*_mol_). The specific spectral absorption coefficients of each *Z*‐isomer are calculated according to Formula (3).

By the analytical C30‐HPLC‐PDA‐ELSD method, the $A_{1\text{cm}}^{1\%}$ of Fraction I (identified as 13 *Z*‐isomer)=1,119 (at λ_max_ = 466 nm), the $A_{1\text{cm}}^{1\%}$ of Fraction II (identified as 9 *Z*‐isomer)=2,183 (at λ_max_ = 468 nm), the $A_{1\text{cm}}^{1\%}$ of Fraction III (identified as all *E*‐isomer)=3,438 (at λ_max_ = 476 nm), and the $A_{1\text{cm}}^{1\%}$ of Fraction IV (identified as 5 *Z*‐isomer)=3,422 (at λ_max_ = 475 nm).

According to Equation(2), the molar absorption coefficient of each *Z*‐isomer is calculated as follows: the *ε*_mol_ of Fraction I = 60,076, the *ε*_mol_ of Fraction II = 117,199, the *ε*_mol_ of Fraction III = 187,260, and the *ε*_mol_ of Fraction IV = 183,717.

### 3.6 Separation and identification of geometrical isomers on preparative C30‐HPLC‐PDA

**FIGURE 6:**
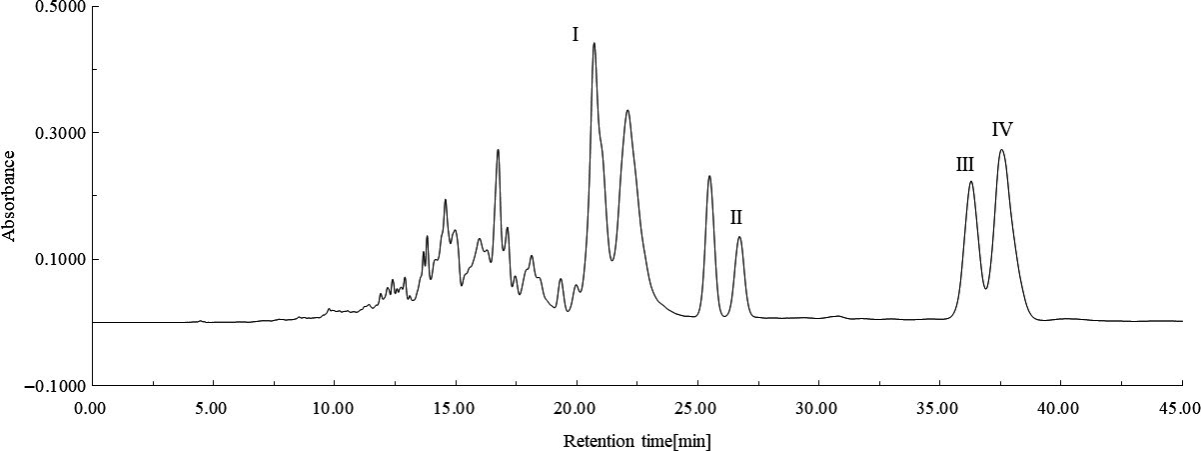
Preparative C30‐HPLC profile of iodine‐induced lycopene geometrical isomer mixture. HPLC conditions: as described in Section 1.2.3. Monitoring wavelength: 476 nm. Fraction identifications: I = 13 *Z*‐isomer; II = 9 *Z*‐isomer; III = all *E*‐isomer; IV = 5 *Z*‐isomer

The preparative C30‐HPLC profile of the geometrical isomer mixture is shown in Figure 6. The fraction identification was undertaken as same as that performed on the analytical column. It was observed that lycopene geometrical isomers from the mixture were well separated on a preparative column under conditions employed in this investigation, while each isomer fraction had a longer retention time in comparison with that on an analytical column.

### 3.7 Peak area integration on preparative C30‐HPLC and fraction amount measurement

The peak area of all *E*‐isomer, and 5, 9, and 13 *Z*‐isomers was integrated after each injection. The total peak area for 70 injections was finally calculated. It was seen that the electronic absorption spectral characteristics of each isomer remained identical, while less than 1% decrease in both peak area and fraction amount of each isomer was seen in over 70 repeated injections. Those observations suggested that the molecular structure, especially geometrical configuration, of each isomer was not changed and the loss of isomer amount was limited.

### 3.8 *Z*‐isomer absorption coefficient calculation on preparative C30‐HPLC

From the $A_{1{\text{cm}}_{\text{AllE}}}^{1\%}$ value of all *E*‐isomer in relation to Equation (1), the $A_{1\text{cm}}^{1\%}$ and *ε*_mol_ values of each *Z*‐isomer are calculated by Equation (4). By the preparative C30‐HPLC‐PDA and gravimetric method, the $A_{1\text{cm}}^{1\%}$ of Fraction I (identified as 13 *Z*‐isomer)=1,116 (at λ_max_ = 466 nm), the $A_{1\text{cm}}^{1\%}$ of Fraction II (identified as 9 *Z*‐isomer)=2,181 (at λ_max_ = 468 nm), the $A_{1\text{cm}}^{1\%}$ of Fraction III (identified as all *E*‐isomer)=3,438 (at λ_max_ = 476 nm), and the $A_{1\text{cm}}^{1\%}$ of Fraction IV (identified as 5 *Z*‐isomer)=3,421 (at λ_max_ = 475 nm).

With Equation (2), the *ε*_mol_ of each fraction is calculated as follows: the *ε*_mol_ of Fraction I = 59,915, the *ε*_mol_ of Fraction II = 117,091, the *ε*_mol_ of Fraction III = 187,260, and the *ε*_mol_ of Fraction IV = 183,663.

It was observed that the $A_{1\text{cm}}^{1\%}$ and *ε*_mol_ values of each *Z*‐isomer by preparative C30‐HPLC‐PDA were slightly lower than those by the analytical C30‐HPLC‐PDA‐ELSD due to the loss of fractional amounts in over 70 injections.

Data from those investigations suggested that the closer the *Z*‐isomerization position was to the geometrical center of the molecule, the smaller the *Z*‐isomer absorption coefficient was. This is fundamentally due to the variation in 1^1^Ag—1^1^Bu transition energy of the molecule.

### 3.9 Scope for future

The variety and nature of solvents have an influence on the $A_{1\text{cm}}^{1\%}$ and *ε*_mol_ values of lycopene geometrical isomers [18]. In theory, the values of each *Z*‐isomer measured can only be used in electron absorption spectroscopy in the mobile phase applied in this investigation. In other mediums including single or mixed solvents, the $A_{1\text{cm}}^{1\%}$ and *ε*_mol_ of each *Z*‐isomer can be deduced from those determined in this investigation.

An increased demand for quantitative analysis of lycopene geometrical isomers is seen in life science, food quality control, animal and human metabolism, and pharmacology fields. It would be expected that the $A_{1\text{cm}}^{1\%}$ and *ε*_mol_ of *Z*‐isomers determined in this study could be applied for *Z*‐isomer quantification before their commercially available reference samples appeared in market.

## CONFLICT OF INTEREST

The authors declare no competing interests.
